# Transcriptome Analyses Reveal the Role of Light in Releasing the Morphological Dormancy of Celery Seed by Integrating Plant Hormones, Sugar Metabolism and Endosperm Weakening

**DOI:** 10.3390/ijms231710140

**Published:** 2022-09-04

**Authors:** Han Li, Jingbo Chen, Lizhong He, Hongfang Zhu, Zhiwu Huang, Minfen Zhu, Linhua Fan, Lingyun Wu, Li Yu, Weimin Zhu, Jun Yan

**Affiliations:** 1 Shanghai Key Lab of Protected Horticultural Technology, Horticulture Research Institute, Shanghai Academy of Agricultural Sciences, Shanghai 201403, China; 2 College of Agriculture, Nanjing Agricultural University, No. 1 Weigang Road, Nanjing 210095, China; 3 Institute of Botany, Jiangsu Province and Chinese Academy of Sciences, Nanjing 210014, China

**Keywords:** celery seed, light regulation, endosperm weakening, seed germination, transcriptome

## Abstract

Celery seed is known to be difficult to germinate due to its morphological dormancy. Light is the key signal to release morphological dormancy and promote seed germination. However, this mechanism has rarely been studied. We performed physiological, transcriptome analyses on celery seed exposed to light and dark to decipher the mechanism by which light promotes germination of celery seed. The results showed that light significantly enhanced the expression of gibberellin synthesis genes and abscisic acid degradation genes and inhibited the expression of abscisic acid synthesis genes and gibberellin degradation genes. Moreover, gibberellin synthesis inhibitor could completely inhibit the germination capacity of celery seed, indicating that gibberellin is indispensable in the process of celery seed germination. Compared with dark, light also increased the activity of α-amylase and β-amylase and the expression of related coding genes and promoted the degradation of starch and the increase of soluble sugar content, suggesting that light enhanced the sugar metabolism of celery seed. In addition, transcriptome analysis revealed that many genes related to endosperm weakening (cell wall remodeling enzymes, extension proteins) were up-regulated under light. It was also found that light promoted the accumulation of hydrogen peroxide in the radicle, which promoted the endosperm weakening process of celery seed. Our results thus indicated that light signal may promote the release of morphological dormancy through the simultaneous action of multiple factors.

## 1. Introduction

Celery (*Apium graveolens* L.) is a widely cultivated horticultural crop and has excellent medicinal value [1]. Celery seed has been studied as an ideal model of morphological dormancy, that is, dormancy caused by underdeveloped (small) embryos embedded in endosperm tissue after harvesting or natural shedding [2,3,4]. In addition to this, celery seed germination faces the double mechanical limitation of pericarp-testa layer and endosperm. Due to the existence of many limiting factors, difficulty in celery seed germination has become an important factor limiting the productivity of celery.

Seed dormancy is the capacity to adapt to the environment acquired in the process of evolution, and the process of dormancy release is coordinated by both internal and external factors [5]. Environmental factors such as light, temperature, and nitrate content in soil may affect the process of dormancy release [6,7]. Internal factors mainly refer to gibberellin (GA) and abscisic acid (ABA), and the stimulatory effect of GA and the inhibitory effect of ABA on seed germination are well documented [8,9,10]. More and more studies indicate that seed germination depends more on the relative content of GA and ABA than absolute content [11]. Plant seeds can be divided into three categories based on their response to white light during seed germination, namely light-requiring seeds, light-neutral seeds, and light-inhibited seeds [12]. Current studies have proved that Arabidopsis and lettuce are light-requiring seeds. When Arabidopsis seeds germinate under light, endogenous ABA content is decreased, which is related to down-regulation of ABA synthesis genes (*NCEDs*) and up-regulation of ABA degradation genes (*CY707A2*) [13]. Meanwhile, GA synthesis genes (*GA3ox*) were up-regulated under light, while the gibberellin degradation genes (*GA2ox*) were inhibited under light [13]. Therefore, light promoted seed germination mainly by affecting the dynamic balance between GA and ABA.

When seeds germinate under suitable conditions, with the growth of radicle, the mechanical restriction of the micropylar endosperm will gradually release, which is called endosperm weakening [14]. It has been reported in early studies that endosperm weakening is involved in promoting the germination of tomato seed [15]. In recent years, it has been found that β-mannanase [16], Xylo-glucan Endo trans-glycolase/hydrolase (XTH), [14] and other cell wall remodeling enzymes (CWREs), as well as hydrogen peroxide [14] can be transported for long distances in plants are involved in endosperm weakening in different mechanisms. At present, the mechanism of endosperm weakening can be divided into enzymatic reaction mechanism (CWREs) and non-enzymatic reaction mechanism (reactive oxygen species and expansion proteins), but related research on the endosperm weakening of celery seed is still rare.

At present, it is known that celery seed belongs to the light-requiring category. Jacobsen found that light can significantly promote celery seed germination and embryo growth, but dark has the opposite effect [17]. Pressman’s research found that under the condition of 25 °C, daily continuous light can significantly increase the germination rate of cultivated celery and wild celery to more than 80% [18]. We know that light is needed for celery seed germination through previous studies, but most studies only used light as a factor to promote celery seed germination and then studied other biological problems. Thus, the mechanism of light releasing the morphological dormancy of celery seed remains to be explored. In addition, light intensity and photoperiod are important parameters to convey the state of external light environment. Nevertheless, the response of celery seeds to different combinations of light intensity and photoperiod has not been reported. To solve this challenge, this study used dark treatment and different combinations of photoperiod and light intensity to explore the effect of different light environments on celery seed germination and used transcriptome to analyze the regulatory mechanism of light on celery seed germination. Our results deepen our understanding of plant acclimation to light environment and provide valuable information for the release mechanism of morphological dormancy.

## 2. Results

### 2.1. Effects of Different Light Intensity and Photoperiod on Seed Germination of Celery

**Figure 1:**
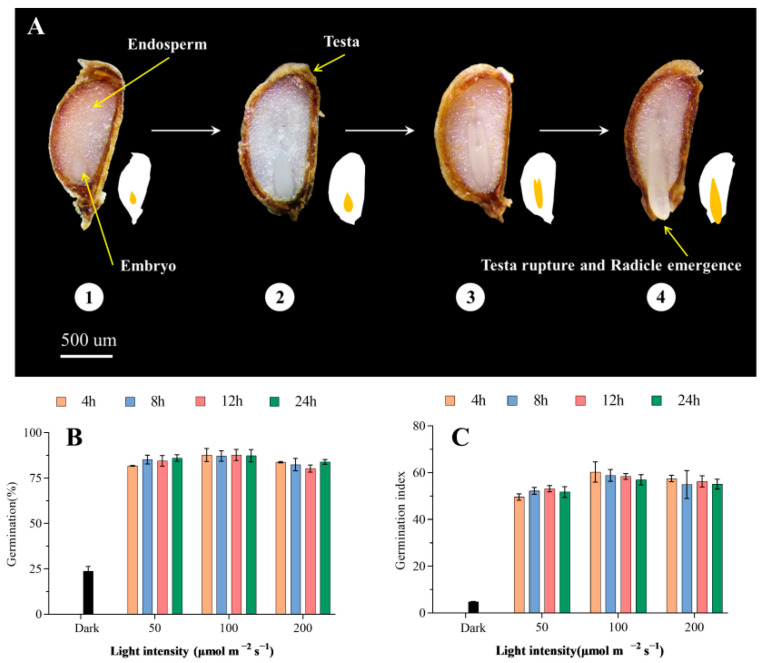
Effects of dark, different light intensity, and photoperiod on germination of celery seed. (**A**) Phenotypes of celery seeds under 100 μmol m^−2^ s^−1^ continuous light for 1, 2, 3, 4 days. (**B**) Germination rate of celery seed under dark, different light intensity (50, 100, 200 μmol m^−2^ s^−1^) and different photoperiod (4, 8, 12, 24 h) (**C**) Germination index of celery seed under dark, different light intensity (50, 100, 200 μmol m^−2^ s^−1^) and different photoperiod (4, 8, 12, 24 h). Vertical bars indicate means ± SD (*n* = 3).

As shown in Figure 1B and Table 1, the germination rate was only 23.8% under dark, while under light, the germination rate was over 84% (Table 1). The germination rate at 100 μmol m^−2^ s^−1^ was significantly higher than that at 50 μmol m^−2^ s^−1^ and 200 μmol m^−2^ s^−1^, reaching 87.5% (Table 1). Compared with light intensity, photoperiod and the interaction between light intensity and photoperiod (*LI* × *P*) had no significant effect on the germination rate (Table 1). In addition, it was observed that light intensity (*η*^2^ = 0.62) had the greatest effect that contributes to the variability of germination index, followed by light intensity×photoperiod and photoperiod (Table 1). Compared with 100 μmol m^−2^ s^−1^ and 200 μmol m^−2^ s^−1^, the germination index decreased markedly at 50 μmol m^−2^ s^−1^ (Figure 1C, Table 1). The above results show that light can significantly improve the germination capacity of celery seed, and the light intensity has a more obvious effect on the germination of seed than the photoperiod. Under the light treatment of 100 μmol m^−2^ s^−1^, the germination rate and germination index were the highest, while under 50 μmol m^−2^ s^−1^, although the germination rate reached 84.4%, the germination index decreased significantly.

### 2.2. Transcriptome Profiling and Identification of Differential Genes 

**Figure 2:**
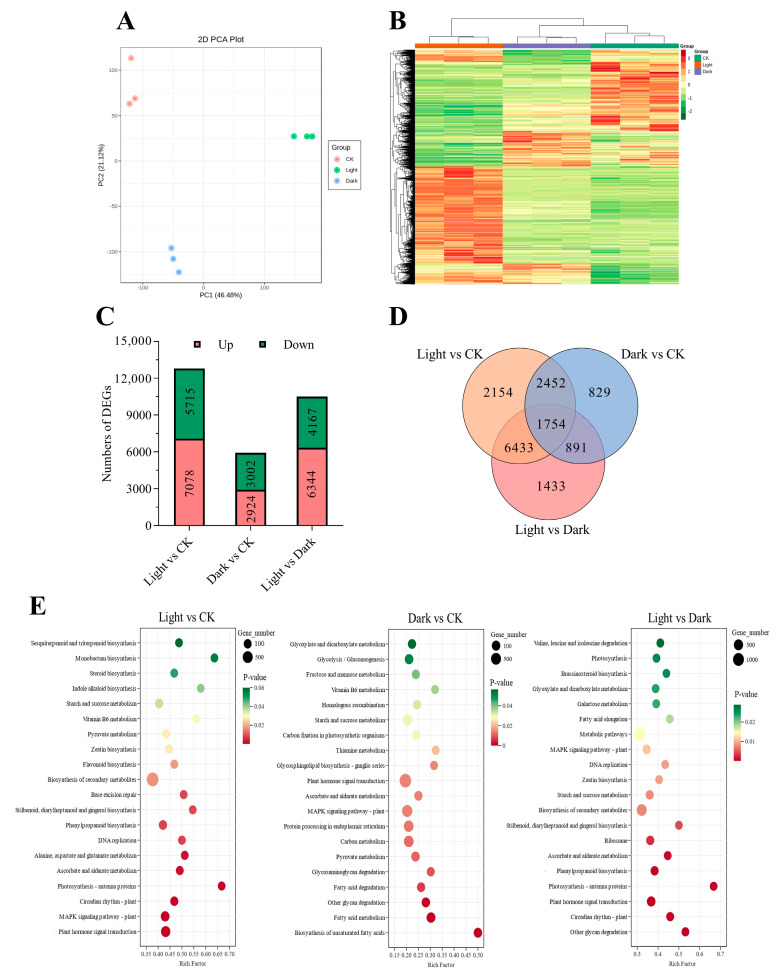
Multivariate statistical analysis of transcriptome data. (**A**) PCA score plot. (**B**) Cluster analysis. The color indicates the relative levels of genes from low (green) to high (red). (**C**) Numbers of genes with differences in relative abundance in different comparison groups. Red and green columns represent the numbers of genes with increases or decreases in relative abundance, respectively. (**D**) Venn diagram showing the overlapping and unique DEGs in the comparison group “Dark/CK”, “Light/CK”, and “Light/Dark”. (**E**) KEGG pathway analysis of DEGs in “Dark/CK”, “Light/CK”, and “Light/Dark”.

To further elucidate the mechanism of light promoting celery seed germination of celery seed, transcriptome analysis was conducted on seeds treated with light, dark, and CK. After removing low-quality reads, the original reads and clean reads produced by each library ranged from 45.06 million to 56.16 million and 40.29 million to 48.49 million. The contents of Q30 and GC were 94.31–98.53 and 43.48−46.54%, respectively, indicating high quality of transcriptome sequencing data (Table S1). PCA (Figure 2A) and clustering heatmap (Figure 2B) showed high repeatability between different treatments. In total, 12,793 (Light/CK), 5926 (Dark/CK), and 10,511 (Light/Dark) different expressed genes (DEGs) were identified in the comparison between different groups. Except for “Dark/CK”, there were more up-regulated DEGs in both “Light/CK” and “Light/ Dark” than down-regulated DEGs (Figure 2C). As can be seen from Venn diagram, unique DEGs in “Light/CK” and” Light/Dark” are 2154 and 1433, respectively, while only 829 DEGs were identified in “Dark/CK” (Figure 2D, Table S2). 

To gain insight into biological processes in response to light and dark, we analyzed the Kyoto Encyclopedia of Genes and Genomes (KEGG) enrichment of all DEGs. Figure 2E shows the top 20 KEGG pathways in the three comparison groups (Table S3). The results showed that there were significant differences in the enrichment pathways of different comparison groups. Several pathways related to fatty acid metabolism and sugar metabolism were markedly enriched only in “Dark/CK”, including “fatty acid metabolism”, “fatty acid degradation”, “fructose and mannose metabolism”, etc. The enriched pathways in “Light/CK” and “Light/Dark” are more similar, including “circadian rhythm-plant”, “phenylpropanoid biosynthesis”, “photosynthesis-antenna proteins”, etc. The pathways present in all three comparison groups included “plant hormone signal transduction” and “starch and sucrose metabolism”.

### 2.3. Expression Differences of Plant Hormone Metabolism Genes under Light and Dark Conditions

GA and ABA belong to diterpenoids and sesquiterpenoids, respectively, and play key regulatory roles in seed germination of many plants. Studies have shown that in the process of terpenoid synthesis, sesquiterpene precursors and isoprene generate GA precursor geranylgeranyl diphosphate (GGPP) [19]. As significant enrichment for DEGs between “Light/CK” was observed in KEGG pathway related to the “sesquiterpenoid and triterpenoid biosynthesis”, we analyzed transcript abundance of genes GA and ABA metabolism.

**Figure 3:**
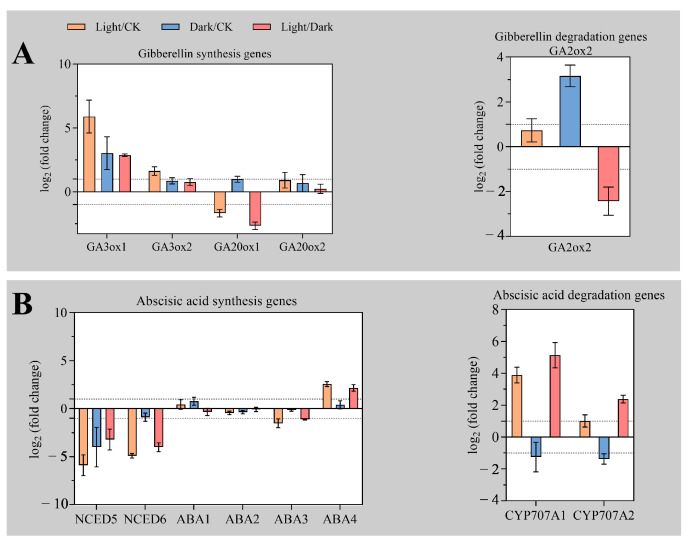
Expression level of plant hormone synthesis and degradation genes. (**A**) Expression level of gibberellin synthesis and degradation genes. (**B**) Expression levels of abscisic acid synthesis and degradation genes. Vertical bars indicate means ± SD (*n* = 3).

The content of GA is affected by both the synthesis and degradation genes. In this study, it was found that *GA3ox1*, *GA3ox2*, *GA20ox1*, and *GA20ox2* genes were up-regulated in “Light/CK”, “Dark/CK”, and “Light/Dark”. The log_2_ (fold change) of *GA3ox1* in “Light/CK” and “Light/Dark” was 5.9 and 2.87, respectively. Therefore, it is speculated that GA synthesis is more intense under light (Figure 3A). GA2ox2 is responsible for the degradation of active GA. This gene was slightly up-regulated in “Light/CK” and significantly down-regulated in “Light/Dark”, but markedly up-regulated in “Dark/CK”. This indicates that compared with dark, the synthesis of GA is activated under light, while the degradation of GA is inhibited (Figure 3A).

ABA plays a key role in maintaining seed dormancy and inhibiting seed germination. In this study, ABA synthesis genes *NCED5* and *NCED6* were significantly down-regulated in “Light/CK”, “Dark/CK”, and “Light/Dark”. In addition, *ABA1* and *ABA2* were not DEGs in “Light/CK”, “Dark/CK”, and “Light/Dark”. *ABA3* was significantly down-regulated in “Light/CK” and “Light/Dark”, but only slightly down-regulated in “Dark/CK”. In contrast to *ABA3*, the transcript abundance of *ABA4* was significantly up-regulated in “Light/CK”, “Dark/CK”, and “Light/Dark”. CYP707A1 and CYP707A2 are responsible for ABA degradation. These two genes have similar expression patterns in “Light/CK”, “Dark/CK”, and “Light/Dark”, which are significantly up-regulated under light and strongly inhibited under dark (Figure 3B). We reasoned that the huge difference in germination rate between light treatment and dark treatment originated from differential metabolism gene expression of GA and ABA.

### 2.4. Effects of Exogenous Gibberellin and Abscisic Acid on Seed Germination of Celery

**Figure 4:**
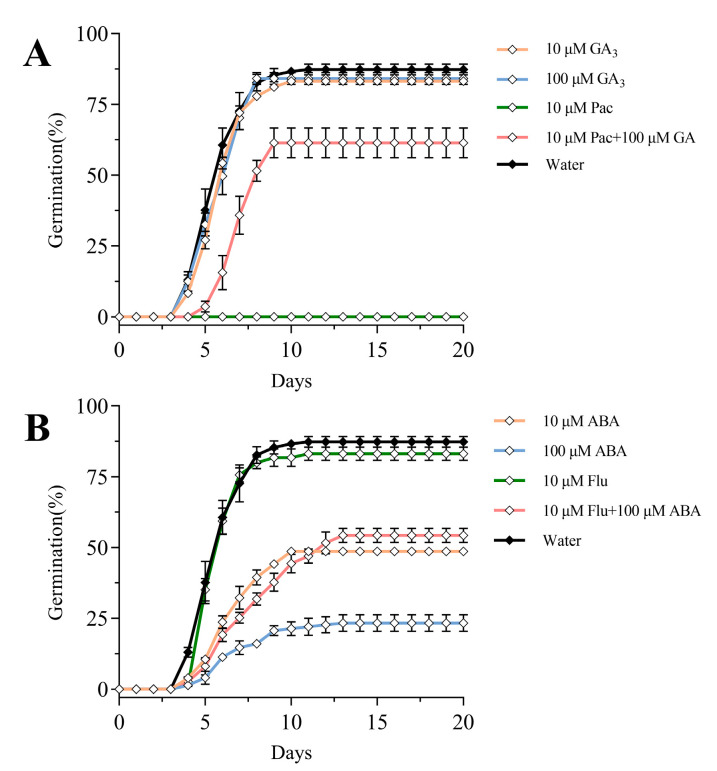
Effects of gibberellin, abscisic acid, and their biosynthesis inhibitors on seed germination. (**A**) Effects of exogenous gibberellin and its synthesis inhibitors on seed germination. (**B**) Effects of abscisic acid and its inhibitors on seed germination. The germination environment of seed was 100 μmol m^−2^ s^−1^, 24 h photoperiod, constant temperature at 24 °C. Both gibberellin and abscisic acid were dissolved by 0.1% DMSO. Vertical bars indicate means ± SD (*n* = 3).

To gain insight into how the celery seed was affected by GA and ABA under light, we utilized a combined approach consisting of exogenous GA and ABA and their synthetic inhibitors. We observed that 10 μM GA_3_ and 100 μM GA_3_ did not appreciably promote germination of celery seed compared with water. However, 10 μM Pac (paclobutrazol, GA synthesis inhibitor) had a profound effect in that it completely inhibited germination capacity of celery seed. Notably, the combined treatment of 10 μM Pac and 10 μM GA_3_ partially restored the germination capacity (Figure 4A). Compared with water, 10 μM ABA significantly inhibited seed germination, and the final germination rate was 48.74%. The inhibition effect of 100 μM ABA was more obvious, and the final germination rate was only 23.3%. Ten μM Flu (ABA synthesis inhibitor) did not significantly promote or inhibit seed germination compared with water, but when 10 μM Flu and 100 μM ABA were used together, the germination rate of celery seed was increased to 54.3% (Figure 4B). These results suggested that GA and ABA were indeed involved in regulating the germination process of celery seed.

### 2.5. Light Increased the Sugar Metabolism of Celery Seed

**Figure 5:**
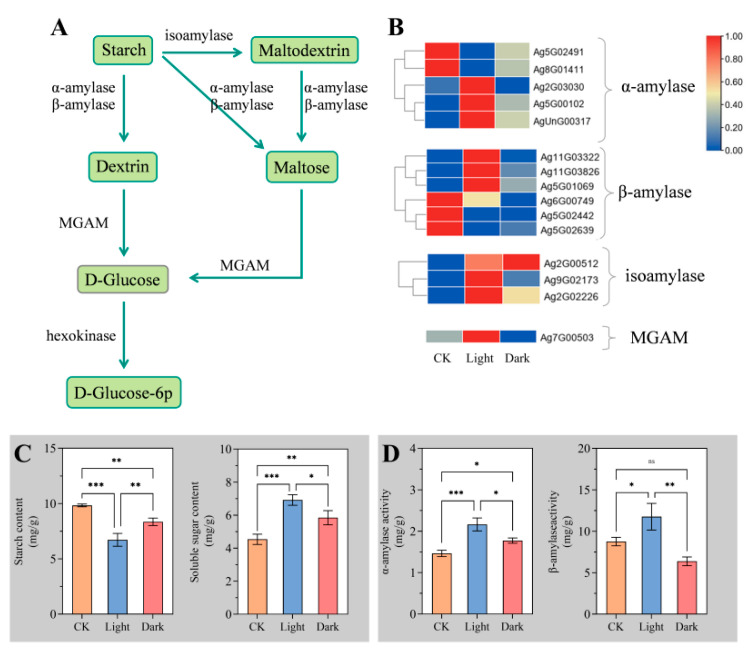
Starch metabolism, sugar content, and amylase activity. (**A**,**B**) Starch metabolism and expression of related metabolic enzyme genes. (**C**) Starch and soluble sugar content. (**D**) α-amylase and β-amylase activity. The transcripts of DEGs were normalized as 0–1. Gene IDs are shown by the legend on the right. Expression levels ranging from red to blue indicate high to low expression for genes, respectively. * *p* < 0.05, ** *p* < 0.01, *** *p* < 0.001, “Tukey’s” test. ns represents no difference. Vertical bars indicate means ± SD (n = 3).

During seed germination, embryo growth mainly depends on starch decomposition and glucose metabolism for energy and carbon skeletons. The starch decomposition process is shown in Figure 5A [20]. Amylase mainly consists of α-amylase and β-amylase, which are involved in the decomposition process of starch to maltodextrin, maltose, and dextrin [21]. In addition, isoamylase and maltase–glucoamylase (MGAM) are also involved in starch decomposition. In this study, all or most of the encoding genes of several enzymes were appreciably up-regulated under light (Figure 5B). Therefore, we also measured the contents of starch and soluble sugar under different conditions. The results showed that compared with CK, the starch content in dark decreased markedly, while the soluble sugar content increased significantly. Under light, starch content markedly decreased compared with CK and dark, while soluble sugar content increased significantly (Figure 5C). Consistent with a decline in starch content, the activity of α-amylase and β-amylase was strongly increased under light (Figure 5D). These results indicated that light improved the sugar supply capacity of celery seed and provided sufficient energy for embryo growth.

### 2.6. Enzymatic Reaction Mechanism of Endosperm Weakening

**Figure 6:**
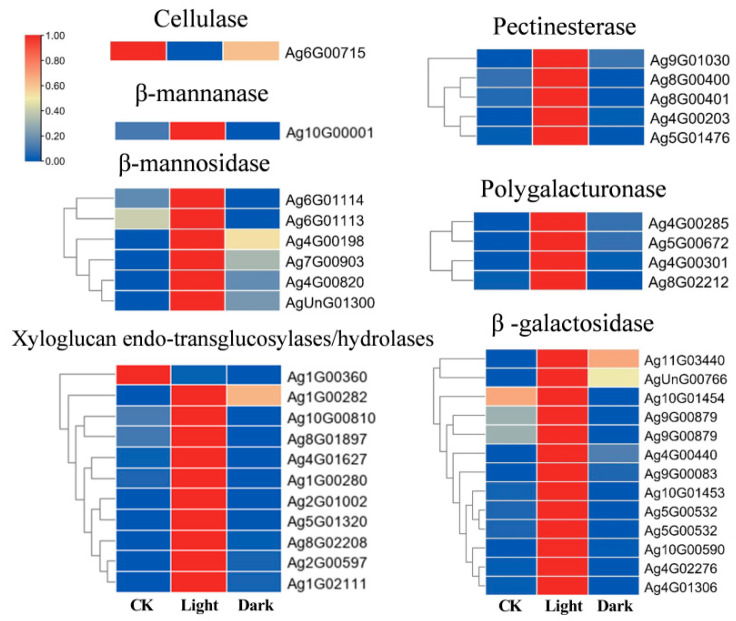
Heatmap visualization of the expression changes of the DEGs related to cell wall remodeling enzymes (CWREs). The transcripts of DEGs were normalized as 0–1. Gene IDs are showed by the legend on the right. Expression levels ranging from red to blue indicate high to low expression for genes, respectively.

It is generally believed that the enzymatic reaction mechanism of endosperm weakening, namely cell wall remodeling enzymes (CWREs), may play an important role in the endosperm weakening process of seed germination. The cell walls of higher plants are composed of cellulose, pectin, and hemicellulose [22]. Enzymes that degrade these cell wall polysaccharides may all play a role in endosperm weakening. Among them, hemicellulose degradation enzymes include xyloglucan endoglycosylase/hydrolase (EC2.4.1.207/3.2.1.151), β-mannanase (EC3.2.1.78), and β-mannoidase (EC3.2.1.25); pectin-degrading enzymes include pectin methyl esterase (EC3.1.1.11), polygalacturonase (EC3.2.1.15), and β-galactosidase (EC3.2.1.23); and cellulose-degrading enzyme identified to cellulase (EC3.2.1.4). The results showed that, compared with CK and dark, all or most of the genes of cell wall remodeling enzymes except cellulase were appreciably up-regulated under light, suggesting that multiple cell wall remodeling enzymes may play a synergic role in endosperm weakening of celery seed (Figure 6).

### 2.7. Non-Enzymatic Mechanisms of Endosperm Weakening

**Figure 7:**
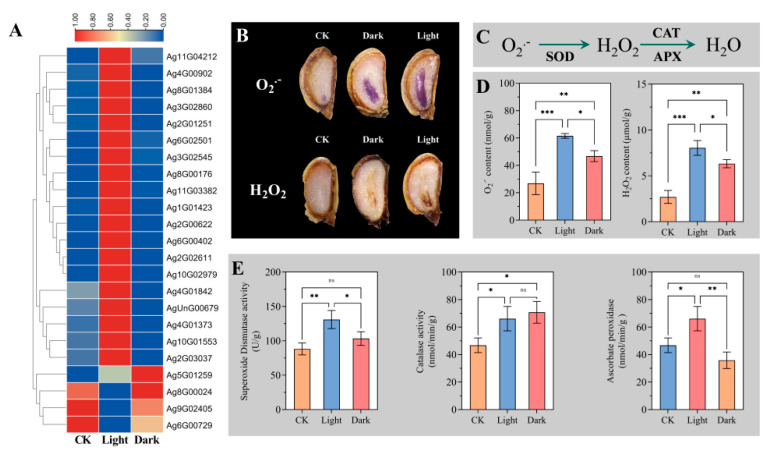
Non-enzymatic mechanisms of endosperm weakening. (**A**) Expression of extension protein genes under different conditions. (**B**) Histochemical staining of O_2_·^–^ and H_2_O_2_. (**C**) Brief steps for reactive oxygen species scavenging. (**D**) Content of O_2_·^–^ and H_2_O_2_ under different conditions. (**E**) SOD, CAT, APX enzymatic activity. The transcripts of DEGs were normalized as 0–1. Gene IDs are showed by the legend on the right. Expression levels ranging from red to blue indicate high to low expression for genes, respectively. * *p* < 0.05, ** *p* < 0.01, *** *p* < 0.001, “Tukey’s” test. ns represents no difference. Vertical bars indicate means ± SD (*n* = 3).

Studies on the non-enzymatic mechanisms of endosperm weakening have focused on expansion proteins and reactive oxygen species (ROS). Interestingly, these transcripts encoding extension protein were up-regulated to a significantly larger extent under light compared with CK and dark (Figure 7A).

To observe the site of ROS biosynthesis, the production and accumulation of O_2_·^–^ were investigated by histochemical NBT staining. The results showed that the whole embryos of CK, light, and dark were stained at the same time, and the degree of staining was relatively uniform. The production and accumulation of H_2_O_2_ were investigated by performing DAB staining, and it was found that CK was not easily stained, dark was slightly stained, and light was more easily stained at the radicle. To further study the role of ROS, the content of O_2_·^–^ and H_2_O_2_ was measured. Compared with CK and dark, the contents of two kinds of ROS in light were significantly increased (Figure 7D). The results shown above suggest that light can promote the production of ROS, and H_2_O_2_ may play a more important role in endosperm weakening.

The ROS content in plants is generally in a dynamic balance. The brief scavenging mechanism of O_2_·^–^ and H_2_O_2_ is shown in Figure 7C. Superoxide dismutase (SOD), Catalase (CAT), and Ascorbate peroxidase (APX) constitute an important part of the scavenging mechanism of ROS [23]. To further characterize the ROS scavenging mechanism, the SOD, CAT, and APX activity were analyzed. It was found that SOD and APX had similar trends after measuring the activity. Compared with CK, light significantly increased SOD and APX activities, while dark had no significant difference with CK. In addition, it was found that both light and dark significantly increased CAT enzyme activity compared with CK, but light and dark showed no significant difference (Figure 7E). 

## 3. Discussion

The main purpose of this study was to screen suitable light environments for the germination of celery seed and uncover the mechanism of light-induced morphological dormancy release combined with transcriptome. Light is not only an energy source for plants but also an important environmental signal. Light signal transmits information about the environment for light-requiring seeds, thus regulating their germination process [24].

In this study, dark conditions did not allow light-dependent seed germination, and the final germination rate of celery seed was only 23.8%. However, the germination rate of celery seed at 50 μmol m^−2^ s^−1^, 100 μmol m^−2^ s^−1^, and 200 μmol m^−2^ s^−1^ all reached at least 84%. The germination index of 50 μmol m^−2^ s^−1^ was significantly lower than that of 100 μmol m^−2^ s^−1^ and 200 μmol m^−2^ s^−1^. However, other studies have found that low light intensity of 25 μmol m^−2^ s^−1^ can significantly promote the germination of light-requiring seeds [25], indicating that light intensity can affect the germination speed and uniformity of celery seed. In contrast, photoperiod had no significant effect on the germination rate and germination index of celery seed (Figure 1B,C, Table 1). Therefore, in this study, 100 μmol m^−2^ s^−1^ full light treatment and total dark were selected for subsequent research, and 200 μmol m^−2^ s^−1^ was not selected for the consideration of energy saving.

Previous studies have shown that light regulates seed dormancy and germination by integrating ABA and GA metabolism and signal transduction [26,27,28]. Light increased GA content and sensitivity of light-requiring seeds, but decreased ABA content and sensitivity [11,29]. In the process of GA synthesis, the inactive form of GA_12_ is synthesized before the active GA, and then GA_12_ is catalyzed by various oxidases to generate GA_1_, GA_3_, etc. with higher biological activity. GA3ox oxidase is responsible for the final step of active GA formation, while GA20ox is responsible for the synthesis of several intermediate inactive GAs [30,31]. In this study, four GA synthesis genes were significantly up-regulated under light. Representatively, the log_2_ (fold change) of *GA3ox1* genes “Light/CK” and “Light/Dark” reached 5.9 and 2.87, respectively. In addition, the GA degradation gene GA2ox2 was more significantly down-regulated under light than in the dark, indicating that light may promote GA accumulation by promoting the expression of GA synthesis genes and inhibiting the expression of GA degradation genes.

The 9-cis-epoxycarotenoid dioxygenase (NCED) is a key enzyme in ABA synthesis [32]. In this study, the expression level of *NCED5* and *NCED6* was markedly inhibited under light. It is noteworthy that ABA4 of ABA family has a higher expression level under light than CK and dark. As shown in Figure S4 [30], the synthesis process of ABA, trans-violaxanthin can be synthesized through two branches, and the high expression of *ABA4* under light may promote the synthesis of Neoxanthin and 9-cis-Neoxanthin. However, *NCED5* and *NCED6* were significantly down-regulated under light, so ABA synthesis might be inhibited during Xanthoxin synthesis by 9-cis-neoxanthin. Dark in contrast to light, ABA synthesis under dark may be more dependent on the branch of 9-cis-violaxanthin.

In the process of seed germination, starch is decomposed into small molecular sugar as a substrate for respiration. Jacobsen et al. observed that starch granules near the radicle are always consumed first [17]. The consumption of starch granules may also reduce the mechanical limitation of radicle growth to some extent. In this study, it was found that the amylase activity and soluble sugar content after light treatment were significantly increased, indicating that light enhanced the sugar metabolism of celery seed and provided more sufficient energy for the growth of the radicle.

β-mannanase has been widely studied in the enzymatic reaction mechanism of endosperm weakening. β-mannanase in Athaliana is composed of eight genes, of which four genes (*AtMAN7*, *AtMAN6*, *AtMAN5*, and *AtMAN2*) are expressed in the endosperm and radicle at the micropylar endosperm before endosperm rupture. Gene knockout experiments show that *AtMAN7*, *AtMAN5*, and especially *AtMAN6* promote the degradation of mannan in Arabidopsis endosperm and accelerate seed germination [33,34]. Carrot and celery belong to Apiaceae family and have the same dormancy type. A β-mannanase gene *DcMAN1* was also identified in carrot seed. The mRNA and β-mannanase activity of *DcMAN1* first appeared in micropylar-half seed, and then appeared in lateral-half seed [35]. In our study, the expression of β-Mannanase gene was significantly increased under light, so it was speculated that β-Mannanase might promote the endosperm weakening of celery seed.

Expansin protein is a kind of cell wall protein that does not have the activity of hydrolase but can break the non-covalent bond (such as hydrogen bond) between cellulose and hemicellulose to relax the cell wall [36]. *LeEXP4* mRNA was specifically expressed in the endosperm region of tomato, and the expression of *LeEXP4* was induced by GA [37]. In our study, most genes of expansin proteins’ family were enhanced by light relative to CK and dark, so it was speculated that expansin protein might be involved in the endosperm weakening process in celery seed.

ROS, especially H_2_O_2_, are byproducts of aerobic respiration [38] and have long been considered as an inhibiting factor for seed germination due to their highly toxic nature to proteins, DNA, and lipids. However, in recent years, the dual role of ROS on seed germination has been gradually recognized. Bailly et al. have proposed that the homeostasis of ROS content affects seed perception of the external environment and acts as an important signal for seed germination [39]. At present, the promoting effect of ROS on seed germination has been confirmed in lettuce [40], rice [41], and Arabidopsis [42]. For example, Zhang et al. found that O_2_·^–^ accumulated in both radicle and microphylla endosperm of lettuce seed during seed germination and could be stained by NBT, while H_2_O_2_ was stained by DAB only in microphylla endosperm [40]. 

However, in this study, it was found that the whole embryo could be stained by O_2_·^–^ after light treatment, while H_2_O_2_ was more likely to be stained at the radicle before endosperm rupture, suggesting that H_2_O_2_ may play a greater role in endosperm weakening. Light also improved the scavenging ability of ROS of celery seed, which kept ROS content in the range of promoting seed germination, thus avoiding the generation of a burst of ROS during the light period (Figure 7B).

## 4. Materials and Methods

### 4.1. Plant Materials and Seed Treatments

The celery varieties used in this experiment are “Shanghai Yellow Heart”. All germination experiments were carried out at a constant temperature of 24 °C. The seeds were soaked in distilled water for 12 h and then placed in petri dishes with two layers of filter paper, supplemented with an appropriate amount of distilled water. Then, the seeds were placed in different light or dark environments and the germination number was recorded every day.

White light was used as the light source in all germination experiments, and the light intensity was 50 μmol m^−2^ s^−1^, 100 μmol m^−2^ s^−1^, 200 μmol m^−2^ s^−1^, photoperiod was 4 h, 8 h, 16, 24 h, and full dark treatment, respectively. The spectrum chart is shown in Supplementary Figure S1. Gibberellin, abscisic acid, paclobutrazol, and fluridone used in the germination experiment in Figure 5 were all dissolved by 0.1% DMSO, and it was verified that 0.1% DMSO had no significant difference in seed germination compared with water (Figure S2). All germination experiments were performed with 50 seeds per replicate. All germination experiments were observed for 20 days. All germination experiments were repeated three times. The germination index was calculated by the following equation [43]:

Germination index = ∑Gt/Dt

Gt is the number of seeds germinated on the day, and Dt is the days of seed germination.

Transcriptome and physiological sampling was performed under the following conditions: (1) CK: Celery seeds soaked in distilled water for 12 h in the dark to saturation, which is the 0th day of germination; (2) Light: After soaking, the seeds were treated under full light conditions for three days (one day before germination) (100 μmol m^−2^ s^−1^, 24 h); (3) Dark: After soaking, the seeds were treated in the dark for three days (one day before germination).

### 4.2. RNA-Seq and Bioinformatics Analysis

Total RNA was extracted using RNAprep Pure Plant kit (DP441, Tiangen, China). The amount of RNA used in each sample was 1 ug, and the first strand of cDNA was synthesized using random primers and M-Mulv reverse transcriptase (RNase H-). The cDNA fragment was enriched and purified after synthesis of the second cDNA strand and splicing and sequenced on Illumina Hiseq X Ten. Clean reads were mapped to the reference genome of celery (http://cgdb.bio2db.com/, accessed on 14 March 2022). Genes with |log_2_foldchange| > 1 and *p* < 0.05 were considered as different expressed genes (DEGs). featureCounts v1.6.2 /StringTie v1.3.4d (https://sourceforge.net/projects/subread/, accessed on 14 March 2022) was used to calculate the gene alignment and FPKM. FPKM is currently the most commonly used method to estimate gene expression levels. KEGG enrichment of DEGs was generated using R based on hypergeometric distribution.

### 4.3. Real-Time Quantitative Reverse Transcription PCR (qRT-PCR)

To further verify the accuracy of transcriptome sequencing results, 10 genes were selected for qRT-PCR validation. Primer3.0 (https://primer3.ut.ee/, accessed on 28 March 2022) was used to design primers (Table S4). Trizol was used to extract RNA from celery seed. cDNA was synthesized using Takara (Takara, Dalian, China) reverse transcription kit. The reaction contained RNA (1000 ng), PrimeScript RT Enzyme Mix I (10 µL), RT Primer Mix (1 µL), 5 × PrimerScript Buffer 2 (for Real Time) (4 µL), and RNase Free dH_2_O was added to obtain a final volume of 20 µL. The qRT-PCR contained 10 µL of TB Green Premix Ex Taq (TaKaRa, Dalian, China), 0.4 µL of forward primer, 0.4 µL of reverse primer, 2 µL of cDNA, and 6.8 µL of ddH_2_O. The relative expression levels of genes were calculated using 2^−∆∆CT^ method. *GAPDH* sequence was used as endogenous control. The results showed that the expression pattern of qRT-PCR results was consistent with that of transcriptome sequencing results, proving the accuracy of transcriptome sequencing results (Figure S3). Three replicates were used for each treatment.

### 4.4. Determination of Sugar Content and Amylase Activity

The soluble sugar content, starch content, α-amylase activity, and β-amylase activity were determined by using kits from Suzhou Comin Bioengineering Co., Ltd. (Suzhou, China). All experiments consisted of three biological replicates.

Briefly, 0.1 g of fresh seeds in 1 mL distilled water were homogenized in an ice bath then placed in a water bath at 95 °C for 10 min. The content of soluble sugar was determined via absorbance measurements at 620 nm. 

For the starch content, 0.1 g of fresh seeds in 1 mL extracting solution were homogenized and then placed in a water bath at 80 °C for 30 min. After cooling, the samples were centrifuged at 3000× *g* for 10 min at 25 °C. The content of starch was determined via absorbance measurements at 620 nm.

For α-amylase determination, a blank group and assay groups were prepared, and 0.1 g of fresh seeds in 1 mL distilled water were homogenized. The homogenate was poured into a centrifuge tube, placed at room temperature for 15 min, and shaken once every 5 min to make it fully extracted. Then, the samples were centrifuged at 8000× *g* for 10 min at 25 °C and diluted with distilled water to 10 mL. The content of starch was determined via absorbance measurements at 620 nm. Total amylase activity and α-amylase activity were measured at the absorbance value of 540 nm. Total amylase activity minus α-amylase activity is β-amylase activity.

### 4.5. Reactive Oxygen Species Content and Antioxidant Enzyme Activity Were Determined

The O_2_·^–^ content, H_2_O_2_ content, SOD activity, CAT activity, and APX activity were determined by using kits from Suzhou Comin Bioengineering Co., Ltd. (Suzhou, China). All experiments consisted of three biological replicates.

Fresh seeds (0.1 g) in 0.9 mL 50 mmol/L phosphate buffer (pH = 7.8) were homogenized in an ice bath and centrifuged at 8000× *g* for 10 min at 4 °C. The content of H_2_O_2_ and O_2_·^–^ was determined via absorbance measurements at 415 nm and 530 nm, respectively.

For SOD, the activity at 50% inhibition in the xanthine oxidase reaction system was defined as one enzyme activity unit (U/g). For CAT, each gram of tissue catalyzing 1 μmol H_2_O_2_ degradation per minute was defined as one unit of enzyme activity. For APX, 1 nmol of ascorbic acid (AsA) per gram of tissue oxidized per minute is 1 unit of enzyme activity (mol/min/g). The changes in absorbance of the reaction solution at 560, 405, and 290 nm per minute were used to calculate SOD, CAT, and APX activity, respectively.

### 4.6. Histochemical Detection of Superoxide Radicals

O_2_.^−^ histochemical staining was performed using Nitroblue Tetrazolium (NBT). The cut half of the seeds were stained in 1 mM NBT solution dissolved with Tris-HCl buffer (pH 7.0) for 40 min, 25 °C, and then washed with buffer and photographed [44].

### 4.7. Histochemical Detection of Hydrogen Peroxide

H_2_O_2_ histochemical staining was performed using DAB (3,3-diaminobenzidine). The halved seeds were stained with 1 mg/mL DAB-HCl (pH 3.8) for 1 h, 25 °C, and then rinsed with water and photographed [45].

## Figures and Tables

**Table 1:** Multivariate analysis of variance results for the effects of light intensity (LI) and photoperiod (P) on germination rate and germination index of celery seed.

Parameter	Estimated Marginal Mean							*LI*		*P*		*LI × P*	
	LI (μmol m^−2^ s^−1^)			P (h)									
	50	100	200	4	8	12	24	*F*	*η* ^2^	*F*	*η* ^2^	*F*	*η* ^2^
Germination (%)	84.4% b	87.5% a	82.6% b	84.4% a	85.0% a	84.2% a	85.8% a	11.5 ***	0.489	0.706	0.081	1.15	0.223
Germination index	51.7 b	56.0 a	58.6 a	55.8 a	55.4 a	56.0 a	54.6 a	19.61 ***	0.62	0.44	0.052	0.89	0.18

Note: The *F* and *η*^2^ were used as measures of *F*-test value and effect size. The *F*-test value (*F*) and effect size (*η*^2^) reflect the effect of model caused by a single factor and the interaction between factors. The *F*-test value is positively related to *η*^2^, and a greater value of *η*^2^ indicates a larger model effect. Different letters in the same row of light intensity and photoperiod columns indicate significantly different levels at “*p*” < 0.05 by “Tukey’s” test. *** *p* < 0.001, Tukey’s test. NS represents no difference. LI: light intensity P: photoperiod.

## Data Availability

The data used in this study to support the findings are available from the corresponding author.

## Supplementary Materials

The supporting information can be downloaded at: https://www.mdpi.com/article/10.3390/ijms231710140/s1.
