# Supplementary figures and images for: Transcriptome Analyses Reveal the Role of Light in Releasing the Morphological Dormancy of Celery Seed by Integrating Plant Hormones, Sugar Metabolism and Endosperm Weakening
Source: Int J Mol Sci. 2022 Sep 4;23(17):10140. doi: 10.3390/ijms231710140 (PMC9456436; doi:10.3390/ijms231710140)

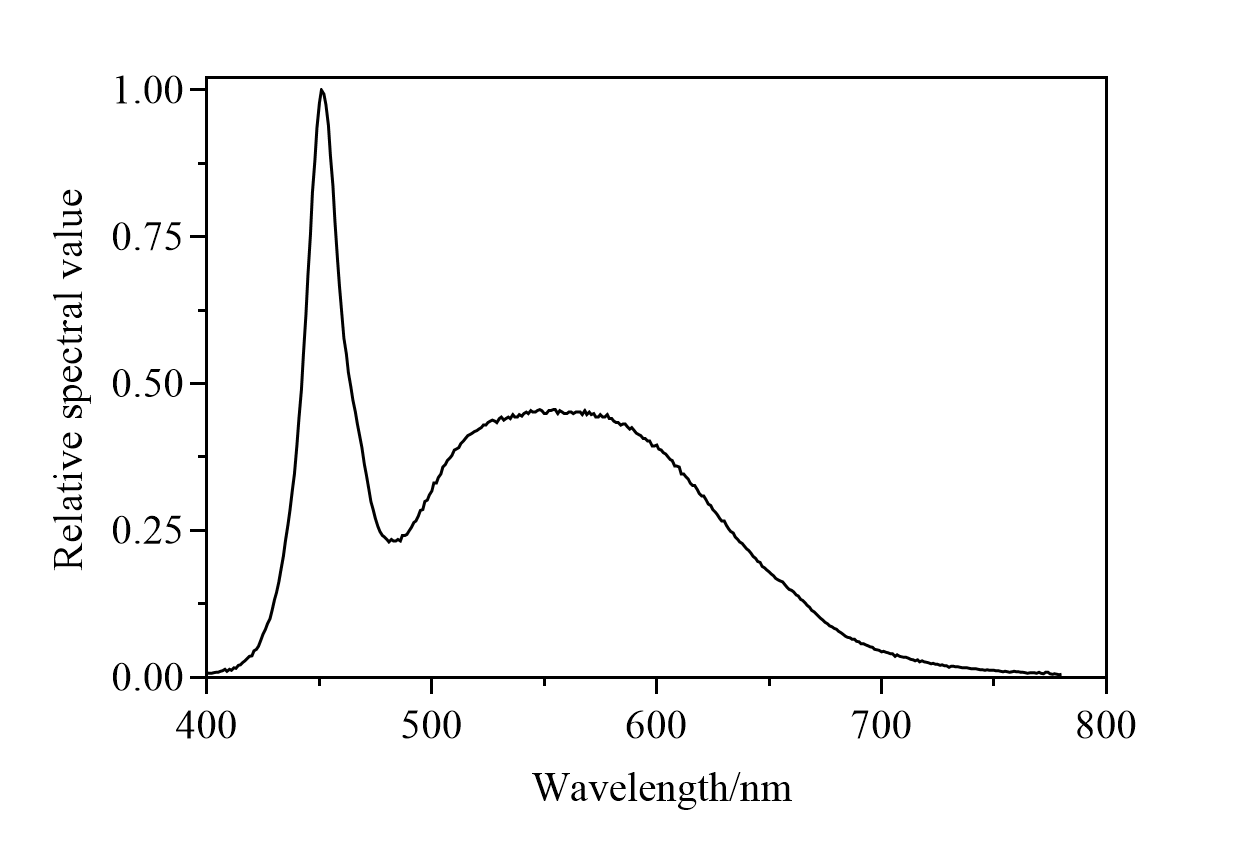

Supplement: Supplementary file 1 [file ijms-23-10140-s001.zip › Figure S1.tif]

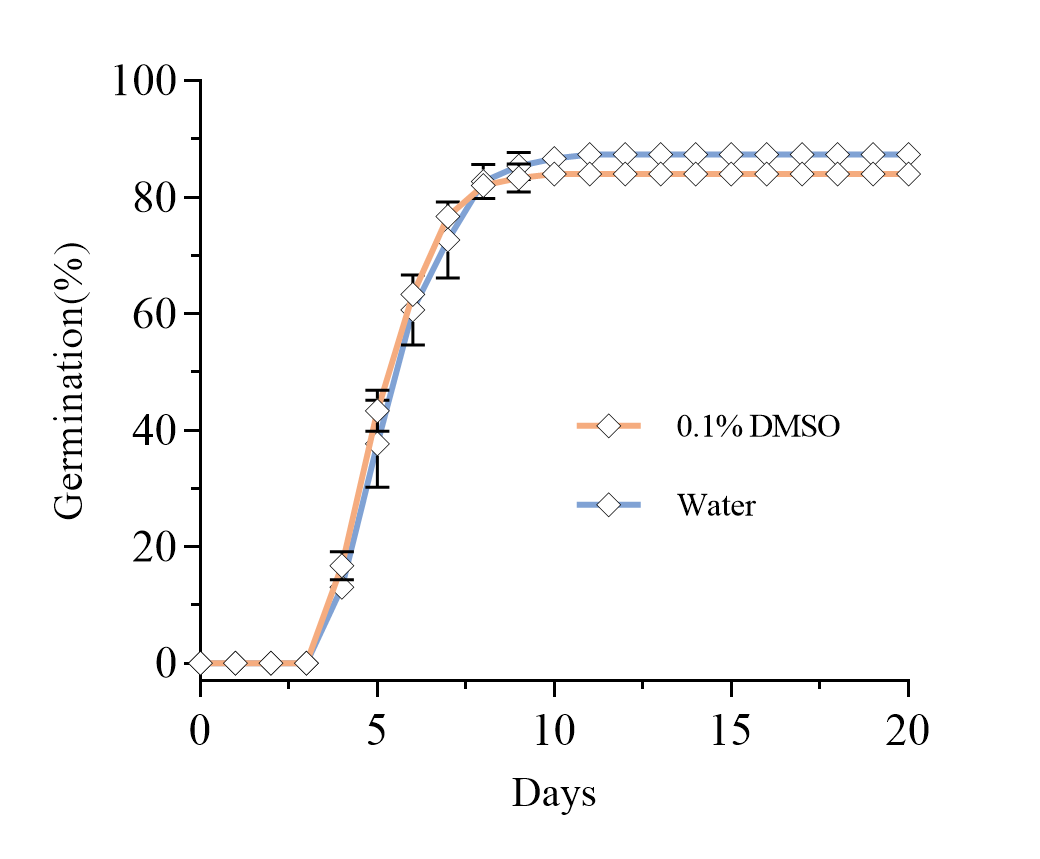

Supplement: Supplementary file 1 [file ijms-23-10140-s001.zip › Figure S2.tif]

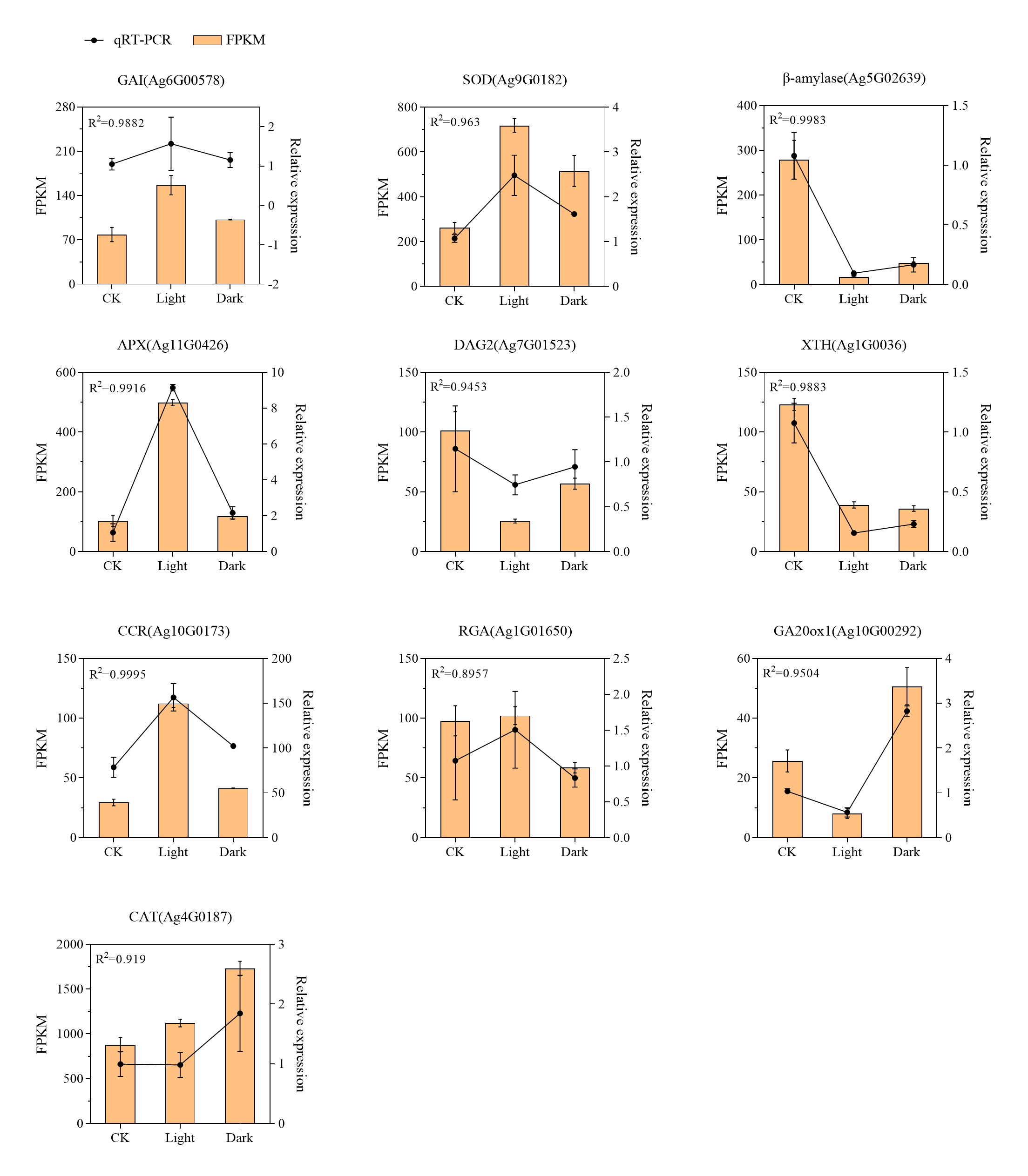

Supplement: Supplementary file 1 [file ijms-23-10140-s001.zip › Figure S3.tif]

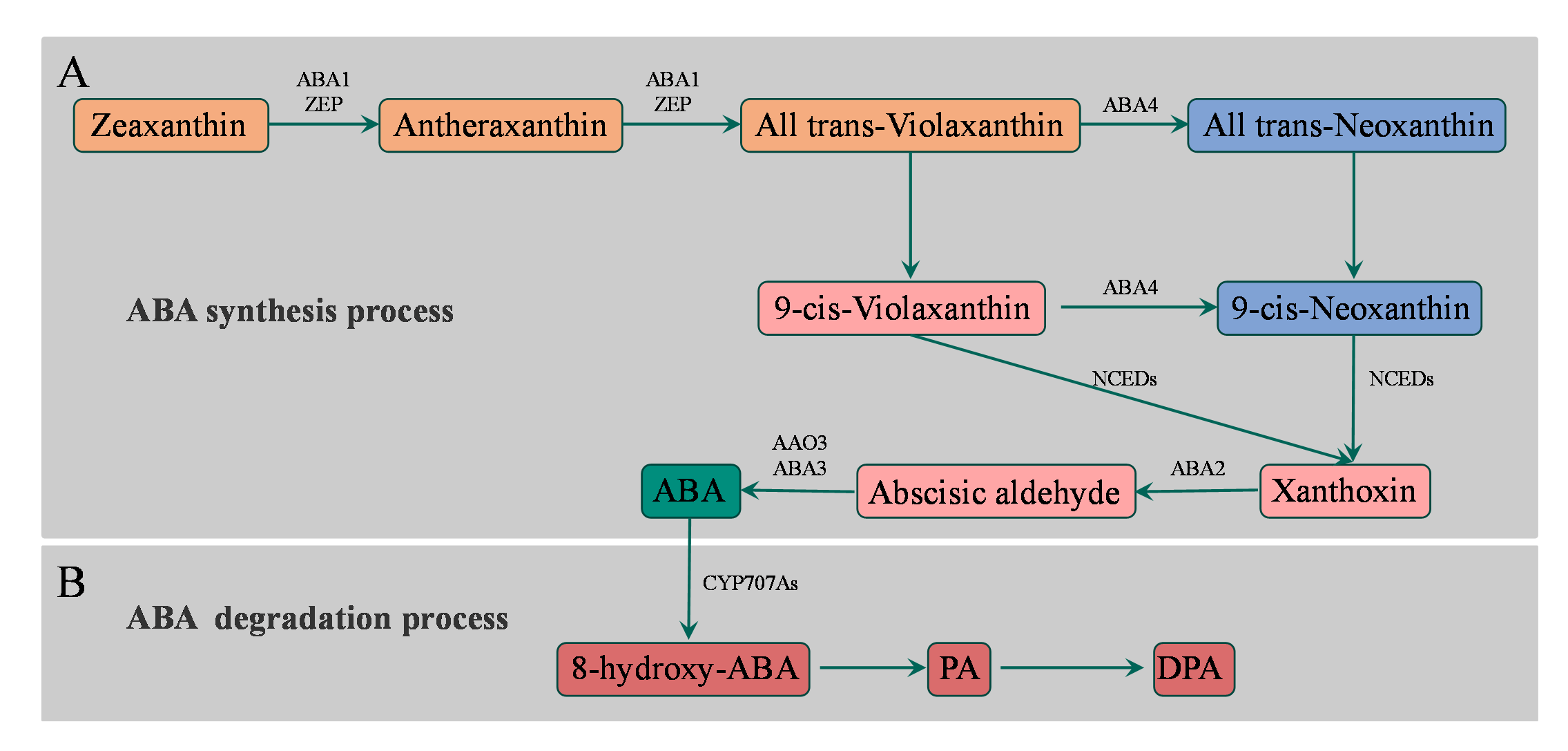

Supplement: Supplementary file 1 [file ijms-23-10140-s001.zip › Figure S4.tif]
